# Amino Acid-Based Protein-Mimic Hydrogel Incorporating Pro-Regenerative Lipid Mediator and Microvascular Fragments Promotes the Healing of Deep Burn Wounds

**DOI:** 10.3390/ijms251910378

**Published:** 2024-09-26

**Authors:** Yan Lu, Shanchun Su, Chih-Chang Chu, Yuichi Kobayashi, Abdul-Razak Masoud, Hongying Peng, Nathan Lien, Mingyu He, Christopher Vuong, Ryan Tran, Song Hong

**Affiliations:** 1 Neuroscience Center of Excellence, School of Medicine, Louisiana State University Health, 2020 Gravier St., New Orleans, LA 70112, USA; ylu@lsuhsc.edu (Y.L.); amasou@lsuhsc.edu (A.-R.M.); nathanlien01@gmail.com (N.L.); cvuon2@lsuhsc.edu (C.V.); rtran@tulane.edu (R.T.); 2 Department of Fiber Science and Apparel Design, Cornell University, Ithaca, NY 14853, USA; 3 Department of Biomedical Engineering, Cornell University, Ithaca, NY 14853, USA; 4 Department of Bioengineering, Tokyo Institute of Technology, Box B-52, Nagatsuta-cho 4259, Midori-ku, Yokohama 226-8501, Japan; 5 Organization for the Strategic Coordination of Research and Intellectual Properties, Meiji University, 1-1-1 Higashimita, Tama-ku, Kawasaki 214-8571, Japan; 6 Department of Environmental Health, University of Cincinnati College of Medicine, Cincinnati, OH 45221, USA; 7 Department of Ophthalmology, Louisiana State University Health, New Orleans, LA 70112, USA

**Keywords:** PreM1: pro-regenerative lipid mediator 1, 14S,21R-dihydroxy-docosa-4*Z*,7*Z*,10*Z*,12*E*,16*Z*, 19*Z*-hexaenoic acid, or 14*S*,21*R*-diHDHA, 3° burn: third-degree burn, AA-PEA: amino acid-based poly(ester amide) polymer, UArg-PEA: unsaturated arginine-based poly(ester amide), ACgel1, UArg-PEA/Chitosan covalently coupled hydrogel 1, MVFs: microvascular fragments, PreM1-MVFs-ACgel1: PreM1-incorporated and MVFs-seeded ACgel1, blood vessel regeneration or vascularization, wound healing, collagen

## Abstract

Pro-regenerative lipid mediator 1 (PreM1) is a specialized pro-resolving lipid mediator that promotes wound healing and regenerative functions of mesenchymal stem cells (MSCs), endothelial cells, and macrophages. The healing of third-degree (3°) burns and regenerative functions of MSCs are enhanced by ACgel1, an arginine-and-chitosan-based protein-mimic hybrid hydrogel. Adipose-tissue derived microvascular fragments (MVFs) are native vascularization units and a rich source of MSCs, endothelial cells, and perivascular cells for tissue regeneration. Here we describe an innovative PreM1-MVFs-ACgel1 construct that incorporated PreM1 and MVFs into ACgel1 via optimal design and fabrication. This construct delivered PreM1 to 3°-burn wounds at least up to 7 days-post-burn (dpb), and scaffolded and delivered MVFs. PreM1-MVFs-ACgel1 promoted the healing of 3°-burns in mice, including vascularization and collagen formation. The re-epithelization and closure of 3° burn wounds were promoted by ACgel1, MVFs, PreM1, MVFs-ACgel1, PreM1-ACgel1, or PreM1-MVFs-ACgel1 at certain time-point(s), while PreM1-MVFs-ACgel1 was most effective with 97% closure and 4.69% relative epithelial gap at 13 dpb compared to saline control. The PreM1-ACgel1 and MVFs-ACgel1 also promoted blood vessel regeneration of 3°-burns although PreM1-MVFs-ACgel1 is significantly more effective. These PreM1- and/or MVF-functionalized ACgel1 have nonexistent or minimal graft-donor requirements and are promising adjuvant therapeutic candidates for treating deep burns.

## 1. Introduction

Severe burn injuries not only significantly diminish patients’ quality of life but also come with an average cost of approximately USD 300,000 per patient [1,2,3,4,5,6,7]. Recognized as a major health concern, severe burns inflict more distress than any other form of trauma [1,2,3,4,5,6,7,8]. Deep burns, and especially third-degree (3°) burns, can destroy full thickness skin, including the epidermis, dermis, and hypodermis, as well as blood vessels [9,10,11,12], leading to scarring (causing morbidity and contracture/disfigurement) [13] and delayed healing [1,2,3,4,5,6,7,8,9,10,11]. Traditional treatment for 3° burns involves skin grafts taken from unburned areas of the patient’s body. However, this harvesting of healthy skin creates additional wounds and can impair function at the donor sites. Moreover, the grafting procedures can be extensive, fraught with risks, expensive, and prone to causing further physical decline. Therefore, the development of a treatment that is safe, user-friendly, and effective in regenerating skin and repairing 3° burns, and that can overcome the drawbacks of currently available treatments, is of paramount importance. Despite the use of current treatment technologies, considerable shortcomings remain in achieving proper wound healing of 3° burns, highlighting the critical need for more efficacious treatment methods [14]. 

Wound healing depends on many factors, including amino acid-based growth factors and cytokines; however, the importance of specific lipid-derived molecules for tissue regeneration in wound healing, resolution of inflammation, and injury repair is now recognized [15,16,17,18,19,20,21,22,23,24,25,26,27,28]. These lipid factors, which include resolvins, neuroprotectins/protectins, maresins, and elovanoids, function as signaling molecules that resolve inflammation and injury, protect against infection, and/or induce the regeneration of cells and tissue [15,16,17,18,19,20,21,22,23,24,25,26,27,28,29,30,31,32,33]. We have identified the molecular structure, biogenesis pathways, and pro-regenerative functions of 14*S*,21*R*-dihydroxy-docosa-4*Z*,7*Z*,10*Z*,12*E*,16*Z*,19*Z*-hexaenoic acid (14*S*,21*R*-diHDHA), a docosahexaenoic acid-derived lipid mediator [16,34]. This small molecule is a specialized pro-resolving mediator (SPM) similar to resolvins and protectins/neuroprotectins, as it is produced by macrophages and possesses inflammation- and wound-resolving properties. 14*S*,21*R*-diHDHA is synthesized by conversion of the essential fatty acid docosahexaenoic acid through sequential catalysis involving 12-lipoxygenase and cytochrome P450, including CYP2E1 [15,16,34]. 14*S*,21-diHDHA has been shown to accelerate wound closure, re-epithelialization, granulation tissue growth, and blood vessel regeneration in murine excisional wounds and to promote the regenerative functions of mesenchymal stem cells (MSCs) in the restoration of wound healing ability [16] and recovery of renal injury [17]. It also promotes microvascular endothelial cell angiogenesis, including migration, tubulogenesis, and production of vascular endothelial growth factor (VEGF) [16,34], suggesting that this molecule recruits endothelial cells to wounds and stimulates the vascularization necessary for the provision of essential nutrients and O_2_ to the newly forming neodermis [15,16]. Based on these functions, we have given 14*S*,21-diHDHA the name **p**ro-**re**generative lipid **m**ediator 1 (PreM1). 

MSCs from adipose tissue or bone marrow show substantial regenerative ability when applied to wounds [1,2,5,35]. Notably, adipose tissue contains **m**icro**v**ascular **f**ragments (MVFs), which are native vascularization units and a rich source of the MSCs, endothelial cells, perivascular cells, and adipocytes needed to rebuild burn-destroyed skin [36,37,38,39,40,41]. MVFs are easily isolated from fat tissue collected by minimally invasive liposuction [36], and compared to isolated MSCs, MVFs provide a physiological niche that boosts MSC regenerative power and promotion of vascular differentiation [36,37,38,39,40,41,42,43]. Sufficient vascularization is a major requirement for the survival and function of both grafted skin/cells and endogenous cells recruited into implanted constructs and burn wounds, as these cells may not survive solely on the O_2_ and nutrients diffusing from nearby tissues [36]. The grafting of MVFs to wounds or implanted constructs has shown promise in supporting grafted and recruited cells because MVFs reassemble into new blood-perfused microvascular networks and develop interconnections with vessels at the host’s wound margin [36,37,38,39,40,41,42,44,45]. 

Unfortunately, in severe burn wound cases, patients with large burns are unlikely to have enough fat tissue to serve as an autologous source of fresh MVFs for use in burn treatment. Furthermore, cultivation to expand MVFs takes many days and is thus unlikely to meet an urgent treatment demand. We hypothesize that PreM1 has the potential to activate small numbers of MVFs to restore burn-destroyed blood vessels and heal deep burns. This would circumvent any need for MSC culture and make clinical treatment of large severe burns feasible using autologous uncultured MVFs at the bedside. This idea is supported by the following: PreM1 induces MSCs and Mϕs to produce VEGF, IGF1, HGF, and/or PDGF, which are key MSC regenerative factors [16,46,47,48]. MSCs injured by oxidative stress show recovery when treated with PreM1, maintain cell viability, and show reduced apoptosis via PI3K signaling [17]. PreM1 treatment of MSCs also markedly promotes the acceleration of blood vessel regrowth and the re-epithelialization of full-thickness excisional wounds [16]. 

The ideal niche for MVF regenerative functions should provide PreM1 (due to the mentioned PreM1 bioactions) and a housing for MVFs. The half-life of PreM1 in wounds is 1 to 2 h, which limits its utility as a functionalizer for MVFs. However, the inclusion of PreM1 in hydrogels (gels) could conceivably provide protection from degradation, while also offering sustained PreM1 release to the wound. Hydrogels, which consist largely of biological fluid and structurally resemble the natural extracellular matrix, can provide three-dimensional (3D) networks that can function as 3D scaffolds for MVFs to promote wound healing [49,50,51,52,53,54,55,56,57,58,59]. In addition, the gels may provide interim replacement of burn-destroyed dermis and promote neodermis growth, while also covering wounds as a conventional primary dressing for burns. 

Hydrogels made from acellular dermal matrices and other natural tissue or organ components, such as collagen and fibrin, have been successfully used to deliver stem cells or other cellular biologics to injury sites, showing promise in promoting repair and regeneration [60,61]. However, these tissue-derived natural biomaterials face challenges such as high cost, reproducibility issues, limited availability, and the potential risk of disease transmission. Therefore, hydrogels fabricated from synthetic biomaterials, which can mimic the beneficial structural, mechanical, and chemical properties of skin, are being explored to overcome these problems [60,62]. In this context, we have further developed a specific prohealing amino acid–based protein-mimic hydrogel [63,64,65,66]. This hydrogel is fabricated by covalent condensation coupling of **u**nsaturated **arg**inine-based **p**oly(**e**ster **a**mide) (UArg-PEA) and a **c**hitosan derivative [glycidyl methacrylate (GMA) chitosan], and is abbreviated as ACgel1 here [63,64]. ACgel1 has excellent biodegradability and biocompatibility without toxicity [64] and is wound-compatible [63]. ACgel1 is able to scaffold and deliver MSCs, promoting accelerated healing of deep burns in mice [63].

The objectives of this study are to develop an ACgel1 incorporated with PreM1 and seeded with MVFs, and to evaluate its release of PreM1 to 3° burn wounds, along with its effectiveness in promoting healing. In this report, we present the fabrication of this construct, its release of PreM1 to 3° burn wounds, and its efficacy in enhancing the healing process. ACgel1 can sustain PreM1 release because of the binding between the anionic PreM1 [15,16] and the cationic Arg and chitosan of ACgel1. The gradual breakdown of this binding, together with UArg-PEA biodegradation, is consistent with our previous reports [63,64]. Mouse 3° burn wounds showed marked promotion of healing when treated with PreM1-containing and MVF-seeded ACgel1. 

## 2. Results

### 2.1. PreM1 Release to 3° Burn Wounds from ACgel1 with and without MVF Seeding

**Figure 1:**
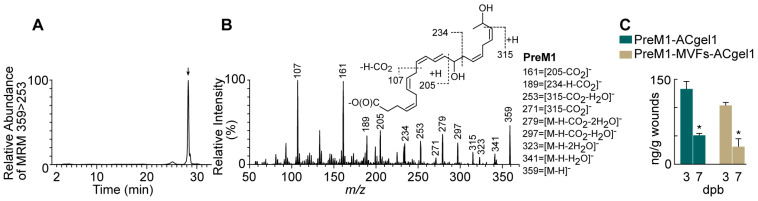
PreM1-containing and MVFs-seeded ACgel1 (PreM1-MVFs-ACgel1) or PreM1-containing ACgel1 (PreM1-ACgel1) delivered PreM1 to 3° burn wounds at least up to 7 days post-burn. (**A**) Typical acLC-MRM [*m*/*z* 359 (Q1) > *m*/*z* 253 (Q2)] chromatogram of PreM1. The arrow marks the peak of PreM1. (**B**) Typical acLC-MS/MS spectrum of PreM1. (**C**) Concentrations of PreM1 in 3° burn wounds delivered by PreM1-ACgel1 or PreM1-MVFs-ACgel1 at 3 or 7 days post-burn (dpb). PreM1 was released from PreM1-ACgel1 that covered the excision-debrided 3° burns that were generated at 0 dpb. Full thickness third-degree (3°) burns were made to the dorsal skin of female C57BL/6 mice and excised at 2 dpb, and then covered with PreM1-ACgel1 or PreM1-MVFs-ACgel1. Wounds and their margins were harvested at 3 or 7 dpb, extracted, and analyzed by acLC-MS/MS for PreM1. Data are means ± SEM; *n* = 3. * *p* < 0.05 versus PreM1 level at 3 dpb.

PreM1 was loaded into ACgel1 networks, and adsorbed by the ACgel1 matrix due to attraction between anionic PreM1 and cationic ACgel1. When PreM1-containing ACgel1 (PreM1-ACgel1) covered and directly contacted wounds, PreM1 was expected to show gradual release into the wounds due to diffusion and the biodegradation of the ACgel1. PreM1 released from ACgel1 in 3° burn wounds at 3 and 7 dpb was authenticated because the chromatogram of aqueous reversed-phase chiral liquid chromatography coupled with tandem mass spectrometry (acLC-MS/MS) multiple reaction monitoring (MRM) (Figure 1A, a typical one) and LC-MS/MS full scan spectrum (Figure 1B, a typical one) acquired from wound extracts matched those of the molecular standard [67]. As illustrated in Figure 1B (insert), the tandem MS/MS spectrum of the chromatographic peak in Figure 1A consisted of ions at *m*/*z* 359 [M-H]^−^, 341 [M-H-H_2_O]^−^, 323 [M-H-2H_2_O]^−^, 297 [M-H-CO_2_-H_2_O]^−^, and 279 [M-H-CO_2_-2H_2_O]^−^, demonstrating two hydroxyls and one carboxyl in the deprotonated molecular ion [M-H]^−^ *m*/*z* 359 (Figure 1B). The 14-hydroxyl was indicated by ions *m*/*z* 205, 234, 189, and 161; and the 21-hydroxyl by ions *m*/*z* 315, 271, and 253, which resulted from the breakage of the C20-C21 bond in the deprotonated molecular ion *m/z* 359 [M-H]^−^ and a neutral loss of CH(O)CH_3_ (44 au) from the terminal group of carbons at the tail of the molecular ion (Figure 1B). PreM1 release into 3° burn wounds from PreM1-ACgel1 or PreM1-MVFs-ACgel1 was sustained up to 7 dpb, whereas PreM1 was undetectable at 3 or 7 dpb in the saline control group based on quantification via LC-MS/MS-MRM (Figure 1C). PreM1 release was still sustained to bioactive levels in 3° burn wounds from the PreM1-ACgel1 or PreM1-MVFs-ACgel1 at 7 dpb based on prior studies [34], although the levels were lower than those at 3 dpb (Figure 1C). Notably, the PreM1 release to 3° burn wounds was higher from the PreM1-ACgel1 than from the MVF-seeded PreM1-ACgel1 (PreM1-MVFs-ACgel1), although the differences were not statistically significant (Figure 1C).

### 2.2. PreM1-Containing ACgel1 Markedly Enhanced MVF Promotion of Wound Closure and Re-Epithelialization of 3° Burn Wounds

**Figure 2:**
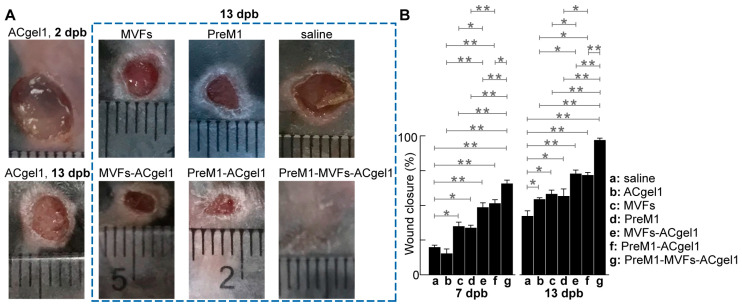
PreM1-containing and MVFs-seeded ACgel1 (PreM1-MVFs-ACgel1) was more effective than an individual or a combination of two from PreM1, MVFs, and ACgel1 in accelerating the closure of 3° burn wounds. (**A**) Representative images of 3° burn wounds with a transparent ruler at the same focal plate as a dimensional reference. (**B**) Wound closure as the percentage of original burn wound area at 7 and 13 dpb. Full thickness 3° burns were made to the dorsal skin of C57BL/6 mice, excision-debrided at 2 dpb, and then treated at 2 dpb with saline (control), ACgel1, MVFs, PreM1, MVFs-ACgel1, PreM1-ACgel1, or PreM1-MVFs-ACgel1. Data are means ± SEM; *n* = 4. * *p* < 0.05 and ** *p* < 0.01.

Treatments with PreM1 [15,16,34,68], ACgel1 [63], or MVFs [36,42,43] individually are already known to promote wound healing. ACgel1 can sustain PreM1 release to burn wounds and promote the healing capacity of MSCs, a key component of MVFs [36,42,43]. However, the effects of the incorporation of PreM1, MVFs, and ACgel1 on wound closure and re-epithelialization of 3° burn wounds were unknown until this study. Treatment of excision-debrided 3° burn wounds with saline control, PreM1 alone, MVFs alone, ACgel1 alone, PreM1-ACgel1, MVF-seeded ACgel1 (MVFs-ACgel1), and PreM1-MVFs-ACgel1 showed that, by 7 days post burn (dpb), the wounds were closed from the initial 6 mm diameter circular full-thickness burns at 0 dpb with closure percentages in the following descending order: MVFs-PreM1-ACgel1, PreM1-ACgel1, MVFs-ACgel1, MVFs, PreM1, saline, and ACgel1 (Figure 2). The wounds treated with PreM1-MVFs-ACgel1 were closed 65.5% on average, which was significantly faster than any other groups (*p* < 0.01), except that of PreM1-ACgel1 (*p* < 0.05) (Figure 2B). Wounds showed significantly greater closure following treatment with PreM1-ACgel, MVFs-ACgel, PreM1, or MVFs than with saline alone (*p* < 0.01 or 0.05), whereas no significant difference was noted between the ACgel1 and saline treatments (Figure 2B). Wound closure was greater following treatment with PreM1-ACgel1 than with PreM1 or ACgel1 alone (*p* < 0.01). Wound closure was significantly greater following treatment with MVFs-ACgel1 than with MVFs or ACgel1 alone (*p* < 0.05 or 0.01).

By 13 dpb, some wounds were closed from the initial burns of 0 dpb. The closure percentages were in the following descending order: PreM1-MVFs-ACgel1, MVFs-ACgel1, PreM1-ACgel1, MVFs, PreM1, ACgel, and saline (Figure 2). The wound closure following treatment with PreM1-MVFs-ACgel1 was 97% on average, which was significantly greater than in any other group (*p* < 0.01) (Figure 2B). Wound closure was significantly greater following treatment with PreM1-ACgel, MVF-ACgel1, PreM1, MVFs, or ACgel1 than with saline alone (*p* < 0.05 or 0.01) (Figure 2B). Wound closure was significantly greater following treatment with PreM1-ACgel1 than with PreM1 or ACgel1 alone (*p* < 0.05). Wound closure was significantly greater following treatment with MVFs-ACgel1 than with MVFs or ACgel1 (*p* < 0.05).

**Figure 3:**
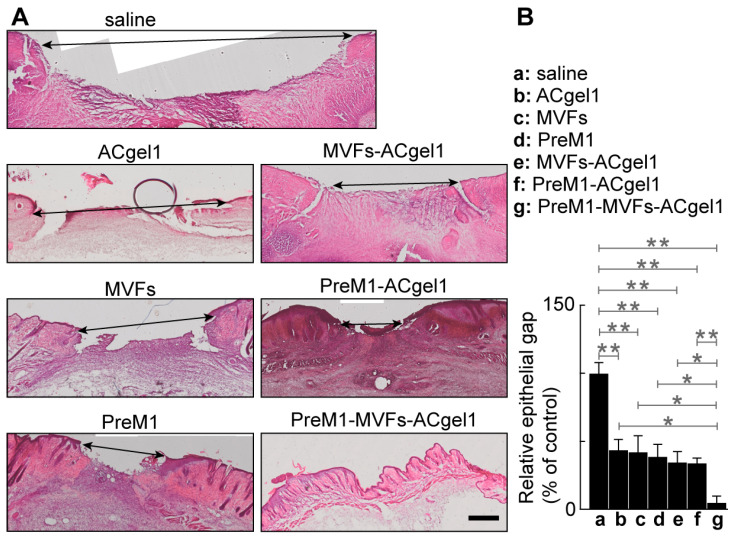
The re-epithelialization of 3° burn wounds was promoted by ACgel1, MVFs, PreM1, MVFs-ACgel1, PreM1-ACgel1, or PreM1-MVFs-ACgel1, while PreM1-MVFs-ACgel1 was most effective. (**A**) Representative microimages of hematoxylin/eosin (H&E)-stained sections of 3° burn wounds with their margins. Scale bar = 500 µm. Arrow-headed lines mark an epithelial gap. (**B**) Relative epithelial gap (% of saline control). Full thickness 3° burns were made to the dorsal skin of C57BL/6 mice at 0 dpb, excision-debrided at 2 dpb, treated at 2 dpb with saline (control), ACgel1, MVFs, PreM1, MVFs-ACgel1, PreM1-ACgel1, or PreM1-MVFs-ACgel1, and collected with margins using a biopsy punch and fine scissors. Wound sections were H&E-stained and analyzed with an Olympus scanning microscope. Data are means ± SEM; *n* = 4. * *p* < 0.05 and ** *p* < 0.01.

Wound re-epithelialization was assessed based on the relative epithelial gap (% of saline control) between neoepithelial edges determined by H&E histochemical analysis of the skin wounds harvested at 13 dpb after euthanasia of the mice (Figure 3). The relative epithelial gaps of 3° burn wounds were significantly reduced by each treatment compared to the saline control, including PreM1-MVFs-ACgel1, PreM1-ACgel1, MVFs-ACgel1, PreM1, MVFs, and ACgel1. Thus, each of these treatments is able to promote re-epithelialization of 3° burn wounds. Moreover, the relative epithelial gap 4.69% of 3° burn wounds treated with PreM1-MVFs-ACgel1 was significantly smaller than in any of the other groups (*p* < 0.05 or 0.01) (Figure 3). Therefore, ACgel1 incorporated with both PreM1 and MVFs is more effective in promoting re-epithelialization of 3° burn wounds than ACgel1 incorporated with just one (either PreM1 or MVFs), and more effective than ACgel, PreM1, or MVFs alone (Figure 3). The data in Figure 2 and Figure 3 demonstrate that ACgel1 incorporated with PreM1 enhanced MVF promotion of wound closure and re-epithelialization of 3° burn wounds.

### 2.3. PreM1 Enhanced MVFs-ACgel1 Promotion and MVFs Enhanced PreM1-Acgel1 Promotion of Blood Vessel Regeneration in 3° Burn Wounds

**Figure 4:**
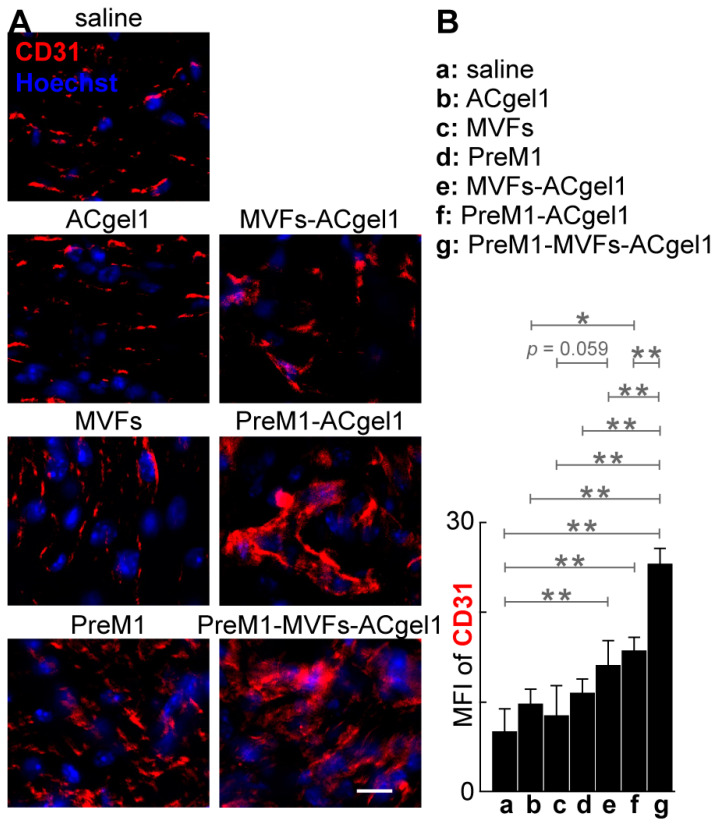
PreM1-containing-and-MVF-seeded ACgel1 was more effective in promoting wound blood vessel regeneration in 3° burns of mice although PreM1-incoporated ACgel1 or MVF-seeded ACgel1 individually was also efficacious. (**A**) Representative microimages of CD31-stained sections of 3° burn wounds of mice. Scale bar is 10 µm. Nuclei were counter-stained blue with Hoechst-33342. (**B**) Quantification of wound vascularity presented as mean fluorescence intensity (MFI) of CD31^+^ vessel areas of 3° burn wounds. Experimental groups were the same as in Figure 3. Immunofluorescent histological analysis using ImageJ was performed to assess CD31^+^ blood vessels in sections (thickness, 10 µm) of 3° burn wounds collected at 13 dpb. Data are means ± SEM (*n* = 4). * *p* < 0.05, ** *p* < 0.01.

To evaluate the treatments on blood vessel regeneration, we determined the CD31+ blood vessels of excision-debrided 3° burn wounds under each treatment by immuno-fluorescent histology (Figure 4). The cryosections of 3° burn wounds possessed significantly higher mean fluorescence intensity (MFI) of CD31+ vascularity (Figure 4), mostly capillary blood vessels (Figure 4A), for the treatment with PreM1-MVFs-ACgel1 (g), PreM1-ACgel1 (f), or MVFs-ACgel1 (e), than for saline control (*p* < 0.01), indicating significantly more blood vessel regeneration of 3° burn wounds under these treatments. Moreover, the wounds had a higher MFI for CD31+ vascularity in the PreM1-ACgel1 group (f) than in the ACgel1 group (b) (*p* < 0.05) (Figure 4B), suggesting that ACgel1 interacts with PreM1 to promote blood vessel regeneration in 3° burn wounds. Additionally, the wounds had higher MFI of CD31+ vascularity for the MVFs-ACgel1 group (e) than for the MVFs group (c), although the significance of difference was marginal (*p* < 0.059) (Figure 4B), which implies that MVFs-ACgel1 could be somewhat more effective than MVFs alone in promoting blood vessel regeneration, and that ACgel1 could interact with MVFs in promoting vascularization in wounds. Furthermore, PreM1-MVFs-ACgel1 (g) was significantly more effective than PreM1-ACgel1 (f) or MVFs-ACgel1 (e) in promoting blood vessel regeneration, as the CD31+ vascularity in wounds was higher for PreM1-MVFs-ACgel1 group (g) than for the PreM1-ACgel1 (f) or MVFs-ACgel1 (e) groups (*p* < 0.01) (Figure 4). This reflects that PreM1 enhanced MVFs-ACgel1 promotion and MVFs enhanced PreM1-ACgel1 promotion for blood vessel regeneration in 3° burn wounds.

### 2.4. PreM1-MVFs-ACgel1 Treatment Increased the Collagen Level in 3° Burn Wounds

**Figure 5:**
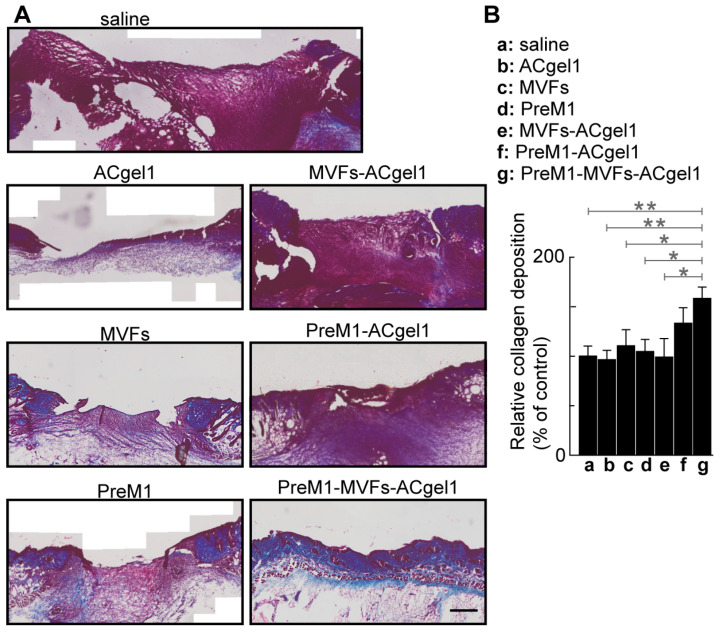
PreM1-containing ACgel1 with MVF seeding increased collagen level in the 3° burn wounds of mice. (**A**) Representative microimages showing Masson-trichrome-stained collagen (blue) in wound sections. (**B**) Relative collagen deposition (% of saline control). Experimental groups were described in Figure 3. Scale bar = 500 μm. Data are means ± SEM (*n* = 4). * *p* < 0.05, ** *p* < 0.01.

Collagen plays an important role in wound healing [69] and skin functions; there-fore, it was evaluated by histochemical analysis in wound sections for each treatment and control. The 3° burn wounds were filled with more collagen, presented as blue Masson-trichrome stain, in the MVFs-PreM1-ACgel1 (g) group than in saline control (a) and ACgel1 (b) groups (*p* < 0.01), in the MVFs (c), PreM1 (d), and ACgel1+MVFs (e) (*p* < 0.05) (Figure 5). Burns treated with PreM1- MVFs-ACgel1 (g) also possessed better-organized collagen and extracellular matrix when compared to wounds treated with saline or other treatments (Figure 5A). The 3° burn wounds of the saline control (a) exhibited poor collagen deposition and an immature extracellular matrix (Figure 5A). These data indicate that the combined incorporation of PreM1, MVFs, and ACgel1 into a novel construct of PreM1-MVFs-ACgel1 can promote collagen formation, which is an important response required for the healing of 3° burns. Notably, the collagen levels in 3° burn wounds were higher when treated with PreM1-ACgel1 or MVFs than with the saline control, although the difference was not statistically significant (Figure 5). 

### 2.5. No Behavior Abnormality Was Observed for Each Treatment Compared to Saline Control

There were no observed differences in mouse behavior, including drinking, foraging, grooming, or eating from 0 dpb to 13 dpb (when mice were euthanized for wound tissue harvesting), between the saline control group and any of the treatment groups (PreM1-MVFs-ACgel1, PreM1-ACgel1, MVFs-ACgel1, PreM1, MVFs, and AC-gel1). This observation suggests that any of treatments was likely to be nontoxic at least for these behaviors.

## 3. Discussion

Severe deep burns are life-threatening and profoundly reduce quality of life [1,2,3,4,5,6,7]. The current clinical treatment for severe deep burns is autologous skin grafts, but these grafts are limited due to donor site unavailability and substantial comorbidities, thereby underscoring the urgent need for more effective therapies [1,2,3,4,5,6,7,8]. The present study was prompted by the existing knowledge regarding wound healing: (1) PreM1 is able to promote wound healing and the regenerative functions of MSCs, endothelial cells, and macrophages; (2) the healing of 3° burns and regenerative functions of MSCs is also enhanced by treatment with ACgel1, our chitosan-coupled amino acid-based polymer hydrogel; and (3) adipose-tissue derived MVFs function as native vascularization units that are enriched with MSCs, endothelial cells, and perivascular cells, and therefore have a high potential to promote the regeneration of deep burn-destroyed skin. Given this background knowledge, we developed an innovative primary hydrogel dressing, PreM1-MVF1-ACgel1, that incorporated PreM1 and MVFs into the ACgel1. In the present study, we tested its utility as a treatment for 3° burn wounds in mice. We observed that PreM1-MVFs-ACgel1, PreM1-ACgel1, MVFs-ACgel1, MVFs, PreM1, or ACgel1 do not cause any immune reactions or inflammation in 3° burn wounds of mice.

The optimally designed and fabricated PreM1-ACgel1 and PreM1-MVFs-ACgel1 constructs were able to deliver PreM1 into 3° burn wounds at approximately 31.5 ng/g of wound tissue up to at least 7 days post-burn when used as a covering that directly contacted the wounds (Figure 1). This level of PreM1 is a prohealing level, as PreM1 promotes angiogenesis of microvascular endothelial cells at the tested dosage range of 1–1000 nM (equivalent to 0.36 to 360 ng/g of wound tissue) [34]. The PreM1 delivered by PreM1-ACgel1 and PreM1-MVFs-ACgel1 was sustained at least up to 7 dpb, although the delivery was only 30–40% of the levels delivered by these two constructs at 3 dpb. Since the half-life of PreM1 in wounds is only a few hours, the PreM1 should be degraded in hours in wounds once it is released into wounds by the PreM1-ACgel1 or PreM1-MVFs-ACgel1. However, the PreM1 delivered by these two gels was sustained at least until 7 dpb as the delivery was continued for at least this period of time. MVFs may degrade PreM1 in the PreM1-MVFs-ACgel1 and/or in the 3° burn wounds, although the amount of degradation by MVFs is unlikely to be significant, since concentrations of the PreM1 delivered by PreM1-ACgel1 and PreM1-MVFs-ACgel1 to the 3° burn wounds were not significantly different (Figure 1C). 

Effective wound closure after deep burns is pivotal for both survival and the best long-term functioning and appearance, as it is needed to prevent infection, fluid loss, and hypertrophic scarring [70], while participating in and supporting other healing processes. Overall, PreM1-MVFs-ACgel1, PreM1-ACgel1, MVFs-ACgel1, PreM1, and MVFs accelerated wound closure at both 7 and 13 dpb, while the degree or quantity of improvement is associated with days post-burn for the observation of wound closure (Figure 2). ACgel1 promoted wound closure at 13 dpb, but this action was not apparent at 7 dpb. The observation that PreM1-MVFs-ACgel1 was the most effective treatment for accelerating wound closure is reasonable because this construct contained three prohealing components, whereas the other treatment groups had only one or two of these components. 

Re-epithelialization is the most crucial response required to rebuild the basic skin barrier in the wound healing process. It is primarily driven by keratinocyte regeneration (migration, proliferation, and differentiation), with participation by epidermal stem cells, fibroblasts, and immune cells [71,72]. Spontaneous re-epithelialization of deep burn wounds progresses slowly, with complete resurfacing in humans often requiring weeks [1,2,3,4,5,6,7,8]. Re-epithelialization and other healing processes are typically induced in deep burns using treatments involving necrotic skin excision and skin grafting [1,2,3,4,5,6,7,8,73]. The aim of the present study was to provide an adjuvant or complementary therapeutic candidate that could reduce or surmount the need for skin grafting. Although all the treatments tested here could significantly accelerate re-epithelialization of 3° burn wounds when compared to the saline control, the PreM1-MVFs-ACgel1 treatment outperformed all the other treatments, likely because it incorporated all three prohealing substances: PreM1, MVFs, and ACgel1. PreM1 delivery to 3° burn wounds was sustained; the sustained release of PreM1 promoted MVF angiogenic and regenerative functions during the healing process while also directly accelerating the healing of 3° burn wounds. These findings were reasonable, because PreM1 is able to promote the angiogenic and regenerative functions of mesenchymal stem cells and endothelial cells, which are both components of MVFs [36,42,43]. 

Blood vessels destroyed by 3° burning must be regenerated to supply oxygen, nutrients, molecular factors, and cells for efficient wound healing and normal skin function [4,5,8]. Our results showed that treatment with PreM1-MVFs-ACgel1, PreM1-ACgel1, or MVFs-ACgel1 accelerated blood vessel regeneration in 3° burn wounds (Figure 4). This finding was consistent with the promotion of wound re-epithelization (Figure 3) and wound closure (Figure 2) by these gels, as the restoration of blood vessels should promote wound re-epithelization and subsequent wound closure. Notably, the 3° burn wounds at 13 dpb appeared to have higher vascularity after treatment with PreM1, MVFs, or ACgel1 compared to the saline control, although the difference was not statistically significant (Figure 4). These treatments could clearly manifest a significant promotion of blood vessel regrowth earlier in the inflammation or proliferation phase, as wound healing is a dynamic process and PreM1 [15,16,34,68], ACgel1 [74], and MVFs [36,42,43] are recognized as prohealing and/or pro-angiogenic factors. This warrants further investigation in the future. 

Another feature of wound healing affected by our treatments was collagen production. Collagen serves both as a construction material during wound healing and as a regulatory signal for the healing process [69]. PreM1-MVFs-ACgel1 treatment increased the collagen levels in the 3° burn wounds, corresponding to its promotion of wound vessel regeneration, re-epithelization, and wound closure. The enhancement of collagen deposition in the 3° burn wounds should increase re-epithelization, wound closure, and wound breaking strength. The PreM1-MVFs-ACgel1 integrates prohealing PreM1, MVFs, and ACgel1 factors so that ACgel1 sustains PreM1 release and scaffolds MVFs for their functioning, while the sustained release of PreM1 promotes MVF functions. Therefore, a logical expectation was that PreM1-MVFs-ACgel1 would outperform treatments consisting of only one or two of these factors in promoting collagen levels in 3° burn wounds. The results confirmed this prediction except that the outperformance by PreM1-MVFs-ACgel1 over PreM1-ACgel1 in promoting collagen levels was not statistically significant. The regenerative or prohealing properties of PreM1, MVFs, and ACgel1 suggest the possibility that PreM1-ACgel1 or other treatments could significantly enhance the collagen levels in 3° burn wounds at later time points when remodeling is further advanced.

## 4. Materials and Methods

### 4.1. Materials

The major chemicals purchased for use in the preparation of ACgel1 included the following: L-arginine (L-Arg), fumaryl chloride, ethylene glycol, and 1,4-butanediol (Alfa Aesar, Ward Hill, MA, USA); chitosan (75–85% deacetylated; molecular weight [MW] 150 kg mol^−1^), and α-isonitrosopropiophenone, 4-(N,N-dimethylamino) pyridine (DMAP, 99%) (Sigma-Aldrich, St. Louis, MO, USA); glycidyl methacrylate (GMA, 97%) (VWR Scientific, West Chester, PA, USA); *p*-toluene sulfonic acid monohydrate and *p*-nitrophenol (J.T. Baker, Philipsburg, NJ, USA); triethylamine (Avantor Performance Materials, Center Valley, PA, USA); Irgacure 2959, Dulbecco’s modified Eagle medium (DMEM), sterile saline (0.9% NaCl or phosphate-buffered saline [PBS]), cell/tissue strainer (500 µm), and solvents (toluene, isopropyl alcohol, N,N-dimethylacetamide, DMSO; ethyl acetate, and acetone) (Thermo-Scientific. Ward Hill, MA, USA). PreM1 (14*S*,21*R*-dihydroxy-docosa-4*Z*,7*Z*,10*Z*,12*E*,16*Z*,19*Z*-hexaenoic acid or 14*S*,21*R*-diHDHA) was prepared through total organic synthesis, and verified and quantified right before its usage for the validation of its structures, purities, and concentrations using our aqueous reversed-phase chiral liquid chromatography coupled with ultra-violet photodiode detector and tandem mass spectrometry (acLC-UV-MS/MS), as described in our previous publications [15,16,34].

### 4.2. Preparation of the PreM1-Containing ACgel1

Following the procedures described in our previous reports [63,64], we cationic prepared ACgel1 from **u**nsaturated cationic **arg**inine-based **p**oly(**e**ster **a**mide) (UArg-PEA) and a cationic **c**hitosan derivative (DS 37 glycidyl methacrylate [GMA] chitosan). We synthesized UArg-PEA via solution polycondensation of Arg alkylene diester monomer salt (I) and the di-*p*-nitrophenyl ester of dicarboxylic acid monomer (II), as we presented in [64], where the synthesis of I and II followed the procedures described in [63,64]. The GMA-chitosan was prepared using our previously described protocol [63,64]. The newly fabricated ACgel1 was soaked overnight in and rinsed thoroughly with deionized water to eliminate unreacted reactant residues. The ACgel1 was pre-cut into cylindrical rods (12 × 6 mm, length × diameter) and soaked in 70% ethanol/water in a tissue culture hood for 20 min for sterilization. The DS 37 GMA-chitosan showed a cationic nature (zeta potential +14.15 to +18.04 mV in aqueous solution at 0.5 mg/mL^−1^).

The rods were then cut into disks (6 mm diameter × 1.5 mm thickness) for subsequent experiments. The 70% ethanol used to soak the ACgel1 disks was replaced with saline (0.9% NaCl) by repeated soaking and rinsing. A 25 µL volume of the saline was then withdrawn from the ACgel1 using a pipette and replaced with 20 µL PreM1 solution (72 µg PreM1/mL in saline, pH 7.4). The solution was allowed to merge into the ACgel1 pores by capillary force and gravity as well as by gently drawing fluid with a pipette from the back side of the ACgel1. The anionic PreM1 was adsorbed by binding to the cationic Arg and chitosan moieties of the ACgel1. 

### 4.3. Animals: General Information

The IACUC committee at the Louisiana State University Health Science Center (LSUHSC) approved all animal procedures, ensuring adherence to the guidelines set by the American Veterinary Medical Association. C57BL/6J mice (Jackson Laboratory, Bar Harbor, ME, USA) were housed at LSUHSC under controlled and specific pathogen-free conditions, with a temperature of 25 ± 2 °C, humidity levels between 50% and 65%, and a consistent 12:12 h light–dark cycle. The mice were fed a standard diet and tap water; both were sterilized. 

### 4.4. Preparation of Adipose Tissue-Derived Microvascular Fragments from Mouse Fat Pads

Microvascular fragments (MVFs) were prepared from the epididymal fat pads of 8–15 month old C57/BL6 male mice under aseptic conditions, according to established protocols [37,38,39,40,41]. Their body weights were more than 30 g/mouse, which provided sufficient amounts of MVFs for AC gel seeding. The mice were anesthetized to the level of no response to a toe pinch using 5% isoflurane inhalation. Their skin surface was sterilized thoroughly with 70% ethanol, and the animals were placed supinely on a sterile drape on a table and immobilized by taping their paws to the surface. The abdominal skin was separated free of the underlying muscle layer by cutting with scissors. We then performed a midline laparotomy with scissors, laterally unfolded the flaps of the abdominal wall, and collected the epididymal fat pads using small scissors and fine forceps. The fat pads were immediately submerged in DMEM (containing 10% fetal bovine serum [FBS], 100 U/mL penicillin, and 0.1 mg/mL streptomycin) at 37 °C. An approximate 3 mm margin was maintained between the epididymis and the fat to avoid unwanted epididymal gathering. The mouse, while still under anesthesia, was then euthanized by cervical dislocation.

The fat pads were rapidly processed after harvesting to minimize potential ex vivo tissue damage. The fat pads were washed with PBS, transferred to 15 mL polypropylene (PP) tubes, and minced with small scissors into a homogenous tissue suspension. The minced fat pads were transferred with two volumes of collagenase type 1 (6 mg/mL PBS, sterile), shaken well, and incubated for 8–15 min at 37 °C and humidified atmospheric conditions with 5% CO_2_ for tissue digestion until the digestate mainly contained “free” MVFs next to single cells. The enzyme reaction was then neutralized by adding 2 volumes of 20% FBS/PBS. We pipetted out the remaining fat, which formed an upper layer due to gravity after incubation (37 °C, 5 min) of the obtained suspension. We repeated this incubation–removal process three times and then removed the remaining fat clots in the suspension by passage through a 500 µm filter. The MVFs in the filtrate were counted using a microscope [38], pelleted by centrifugation (120 g, 5 min), and then resuspended in sterile saline (0.9% NaCl) to 500 MVFs/µL saline for MVF seeding. The final suspension also contained ~9,600 single cells/µL saline. The single cells mainly include endothelial cells, α-smooth muscle actin (SMA)-positive perivascular cells, and MSCs based on the studies by other groups [37,38,39,40,41]. 

### 4.5. MVF Seeding of PreM1-Containing ACgel1

Seeding was performed based on the methods established by others [37,38,39,40,41] and on our method for seeding mesenchymal stem cells to ACgel1 [63]. In brief, 20 µL saline was pipetted out from the PreM1-containing ACgel1 or control ACgel1, followed by the transfer of a 20 µL suspension, containing 500 MVFs/µL saline, to the front side of the gel. The MVFs seeded into the gels during the downward flow of saline carrier due to the capillary force of gel pores, gravity, and negative pressure drawing generated by pipetting at the back side of the gel; any liquid that could be taken by the pipette was added back to the top of the gel.

### 4.6. Mouse Model of 3° Burn Wounds and Treatments

The experimental groups with 3° burn wounds included a saline control, PreM1 alone, MVFs alone, ACgel1 alone, PreM1-containing ACgel1 (PreM1-ACgel1), MVF-seeded ACgel1 (MVFs-ACgel1), and MVF-seeded PreM1-ACgel1 (PreM1-MVFs-ACgel1). Each treatment group included four female C57BL/6J mice aged between 5 and 10 months (body weight: 22 to 25 g/mouse). The mice were acclimated more than a week before the procedure. We removed the hair from the mouse skin where burning was imposed, and the surrounding area, using a hair clipper (https://thecutbuddy.com, accessed on 5 September 2024) followed by hair removal cream (VEET Gel Cream Sensitive Formula, https://www.veet.us, accessed on 5 September 2024). The cream was washed completely from the skin using warm water. We moderately anesthetized the mice to minimize potential distress and mobility with 2% isoflurane inhalation, even though the hair removal procedures are painless. After 6 to 12 h, we anesthetized the mice using 3–5% isoflurane inhalation and administered sustained release buprenorphine (Wedgewood Pharmacy, Swedesboro, NJ, USA) (1 mg/kg, SQ) with analgesic action for up to 3 days.

A mouse model of a third-degree burn of the dorsal skin was employed using the published methodology and device design with modifications [9,10,11,75,76]. Briefly, under sterile conditions, paired, circular, uniform 6 mm full-thickness 3° burn wounds were created symmetrically along the midline of the dorsal skin of each mouse. We generated the two burns by sandwiching the dorsal skin fold of the mouse, at 55 g force perpendicularly to the fold for 40 s, with the flat 6 mm diameter ends of two brass cylindrical rods. The surface temperature of each end that contacted the skin was accurately and rapidly controlled to 110 °C for burning using a digital programmable mini-soldering iron (TS100, GtFPV, Sarasota, FL, USA). We obtained the skin fold by gently holding the full-thickness skin along the midline with our fingers (without stretching, as the skin is loose). The skin was unfolded, returned to the original position immediately after the burn, and then wrapped with Tegaderm waterproof film dressings (www.3m.com, St. Paul, MN, USA, catalog number 16002, accessed on 5 September 2024) for protection and water loss prevention. Each mouse was housed singly in a pathogen-free cage with paper bedding. 

At 48 h or 2 days post-burn (dpb), the mice were anesthetized and provided with analgesia again, as above for burning. We debrided the 3° burns by excising a circle of coagulated or necrotic full-thickness skin 6 mm in diameter from each burned site using a biopsy punch and fine surgery scissors and forceps. This excision resembles the clinical practice of debriding deep burns. We then filled each excision-generated space with a specific 6 mm diameter piece of ACgel1, including ACgel1 alone, PreM1-ACgel1, MVFs-ACgel1, or PreM1-MVFs-ACgel1. For comparison, we injected MVFs (10,000/wound), PreM1 (0.5 nmol/wound/day), or saline vehicle (0.9% NaCl, 20 µL/wound) directly onto the wound margin (5 µL into tissue under the wound bed and 5 µL/site into 3 sites of the dermis along the wound edge). We wrapped the wound areas and the hair-removed skin of the mouse body trunk with Tegaderm films to maintain the position and hydration of the ACgel1 and wounds as well as to minimize wound contraction, as we have done previously [56,77]. This can assist wound closure through re-epithelialization, as described in prior studies [78,79,80], thereby better resembling wound healing in humans.

### 4.7. Postoperative Care, Wound Closure Measurement, and Sampling

Fluid resuscitation was conducted by intraperitoneal injection of 1.7 mL 37 °C sterilized 0.9% NaCl saline within 1 h post-burn and 0.5 mL at 12 h post-burn to a mouse with a body weight ~24 g [74]. The fluid resuscitation was continued at 1.2 mL/mouse/12 h until the mouse regained normal activity. We checked mouse health and behavior, including drinking, foraging, grooming, and eating, and we inspected all wounds twice per day to maintain the ACgel1 and dressing. We determined wound healing as performed previously [15,16,34]. To clearly observe the wounds, we changed the Tegaderm film at 7 dpb. The wounds were photographed with a ruler in the same focal plane as a reference for size measurement. Their areas were determined using ImageJ software (Version 1.53t, National Institute of Health, Bethesda, MA, USA). The wound closure was evaluated as the closed wound area (%) relative to the initial burn-wound area. The mice were euthanized at 13 dpb, and full-thickness wounds with 3–5 mm skin margins were excised and fixed in 4% paraformaldehyde in PBS (pH 7.4, 12 h, ~4 °C on water ice and in refrigerator). 

### 4.8. Determination of PreM1 Release to 3° Burn Wounds from ACgel1 with and without MVF Seeding

The PreM1-MVFs-ACgel1 treatment described in Section 4.6 was also conducted specifically for this study in parallel to those for the histological assessment of wound healing. These mice were euthanized by decapitation with sharp scissors after deep anesthesia via 5% isoflurane inhalation (three mice at 3 dpb and three mice at 7 dpb). A piece of full-thickness skin containing an excision-debrided 3° burn wound treated with PreM1-MVFs-ACgel1 was harvested using a 12 mm diameter circular biopsy puncture and microsurgery scissors and forceps, weighed, and then submerged in ice-cold 80% acetone containing butylated hydroxytoluene (0.005%) as an antioxidant and deuterium-labeled internal standard prostanglandin-D_2_-d_4_ (Cayman Chemical, Ann Arbor, MI, USA), and chilled immediately with liquid nitrogen. Each sample was minced with fine scissors to sub-millimeter pieces, homogenized, and sonicated in the harvesting solvent for extraction, as reported previously [15,16,34]. The extracts were purified via C18-solid-phase extraction and then analyzed using aqueous reversed-phase chiral liquid chromatography tandem mass spectrometry (acLC-MS/MS), as detailed previously [15,16]. The acLC-MS/MS (TQ-S tandem mass spectrometer coupled to an Acquity LC [Waters, Milford, MA, USA]) used a chiral column (Chiralpak-IA, 150 mm × 2.1 mm × 5 μm; Chiral Technologies, West Chester, PA, USA). The mobile phase was pumped at 0.2 mL/min using the following gradient: 73% A (water:acetic acid = 99.99:0.01) + 27% B (methanol:acetic acid = 99.99:0.01) (0–1 min); ramped to 70.8% A + 29.2% B (1–5 min), to 14.6% A + 85.4% B (5–50 min), and then to 100% B (50–51 min); continued as 100% B (51–56 min); and a final return to 73% A + 27% B. The MeOH solutions from wound extracts were injected into the acLC-UV-MS/MS. The effluent of the chiral LC was atomized by electrospray, and the negatively ionized PreM1 from wound extracts was identified and quantified for comparison with a PreM1 standard using the LC-MS/MS system [15,16,68,81].

### 4.9. Histochemical and Immunohistological Studies of 3° Burn Wounds under Different Treatments

The fixed tissue samples were cryoprotected in PBS sucrose gradients (15 and 30%) at 4 °C until the tissue settled at the bottom (usually overnight for each gradient), then embedded into OCT (optimum cutting temperature matrix, Tissue-Tec, Torrance, CA, USA) with their anatomic orientations annotated on the silicone molds, and then stored at −80 °C for histological analysis, as previously described [15,34]. Serial cryosections (10 µm thickness/section) of OCT-embedded wounds were made using a cryostat microtome (RWD Life Science Co., San Diego, CA, USA) and transferred to Superfrost slides. The sections were stained with a hematoxylin and eosin (H&E) kit to determine the wound re-epithelization/epithelial gap, with a Masson’s trichrome stain kit (Tyr Scientific LLC, Austin, TX, USA) to assess the wound collagen deposition (where collagen was stained blue) and remodeling, and with CD31 primary antibody (host: rat, Thermo-Fisher, Boston, MA, USA), secondary antibody (host: goat, anti-rat Alexa Fluro 594 [red], Thermo-Fisher), and Hoechst-33342 (staining cell nuclei blue, Thermo-Fisher) to evaluate wound blood vessel regeneration, as described previously [15,16,34]. The H&E-stained sections were visualized using an OLYMPUS scanning microscope (Olympus BX61VS, Olympus America, Center Valley, PA, USA). The epithelial gap (i.e., the distance between the neoepithelium emerging from the wound edge) was determined using OLYMPUS software (VS-ASW FL 2.5, Olympus America), as described previously [16,34,63]. The capillary vascular density in the wound bed was determined using ImageJ as the mean fluorescence intensity (MFI) of CD31^+^ tissue (showing red fluorescence) in the microscope field of the wound bed in the wound cryosections. 

### 4.10. Statistical Analysis

Statistical analysis was conducted using *t*-tests or ANOVA followed by Tukey’s multiple comparison test via GraphPad Prism 9.0.0 software (Boston, MA, USA), and *p* < 0.05 was considered statistically significant. Data are presented as mean ± standard error of mean (SEM). 

## 5. Conclusions

**Figure 6:**
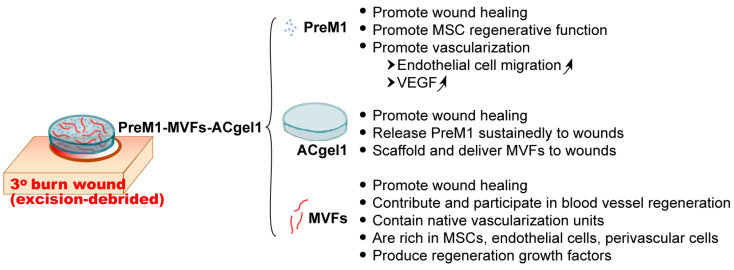
A schematic representation: the mechanism in the literature for actions of each hydrogel component in PreM1-MVF1-ACgel1 for healing 3° burn wounds. The upward-slanting arrow indicates an increasing trend.

This study achieved a novel primary hydrogel-based dressing, PreM1-MVFs-ACgel1, that can effectively promote the healing of 3° burn wounds in mice. PreM1-MVFs-ACgel1 incorporates pro-regenerative lipid mediator 1 and microvascular fragments into an ACgel1 fabricated by covalent condensation coupling of arginine-based poly(ester amide) and a chitosan derivative. Both PreM1-MVFs-ACgel1 and PreM1-ACgel1 can provide sustained release of PreM1 to 3° burn wounds. PreM1-MVFs-ACgel1 accelerated wound closure, re-epithelialization, blood vessel regeneration, and collagen formation in 3° burn wounds. We are currently endeavoring to produce an even more effective modality to heal severe deep burn wounds by the development of other primary hydrogel-based dressings that incorporate different PreM molecules and by assessing PreM1-MVFs-ACgel1 and other gels fabricated using different PreM doses, different MVF doses, and/or different amino acid-based hydrogels. These studies include electron microscopy and mechanical characterizations of these constructs before and after application to wounds at different time points during the healing process; evaluations of the transmigration, proliferation, and expression of key healing-associate factors at protein/peptide and mRNA levels; exploration of the vascularization by MVFs that move from gels to wounds; verification of results using human MVFs in vitro; polarization of macrophages to a reparative phenotype; and resolution of excessive oxidative stress and chronic inflammation in 3° burn wounds. The wound healing mechanism, as well as the mechanisms underlying angiogenesis, and inflammatory modulation during the treatment phase using PreM1-MVFs-ACgel1, should be investigated at molecular and cellular levels in the future. These mechanistic studies will benefit from the mechanism in the literature for actions of each hydrogel component in PreM1-MVF1-ACgel1 that is depicted in Figure 6. The major benefit of this new modality is the reduction or elimination of reliance on graft-donor requirements, meaning that these gels could be promising adjuvant therapies that can overcome the drawbacks of the grafting methods or skin substitutes currently used to treat severe deep burns.

## Data Availability

Data are contained within the article.
